# The Dust Cart, Some Wind, and the Baby

**Published:** 1924-08

**Authors:** 


### The Dust Cart, Some Wind, and the Baby

Sir,—I was held up for a moment the other day at the street corner while a large open dust-cart, full to the brim with refuse, went lumbering by. Between us was a perambulator containing an at-present healthy baby. There was a very fine stiff wind blowing; it blew merrily across the contents of the dust-cart on to the precious content of the perambulator.

Is it of any use to commend to the notice of our local authorities generally the practice of the more enlightened, of having all street and house refuse carted through our streets in covered receptacles? PASSER-BY.

